# Compound heterozygous variants in *PGAP1* causing severe psychomotor retardation, brain atrophy, recurrent apneas and delayed myelination: a case report and literature review

**DOI:** 10.1186/s12883-016-0602-7

**Published:** 2016-05-21

**Authors:** Matthias Kettwig, Orly Elpeleg, Eike Wegener, Steffi Dreha-Kulaczewski, Marco Henneke, Jutta Gärtner, Peter Huppke

**Affiliations:** Department of Pediatrics and Pediatric Neurology, University Medical Center, Georg August University, 37075 Göttingen, Germany; Monique and Jacques Roboh Department of Genetic Research, Hadassah-Hebrew University Medical Center, Jerusalem, Israel

**Keywords:** Whole exome sequencing, *PGAP1* mutations, Neonatal seizures, Leukoencephalopathies, Developmental Disabilities, Case report

## Abstract

**Background:**

Mutations in proteins involved in the glycosylphosphatidylinositol anchor biosynthesis and remodeling pathway are associated with autosomal recessive forms of intellectual disability. Recently mutations in the *PGAP1* gene that codes for PGAP1, a protein localized in the endoplasmic reticulum responsible for the first step of the remodeling of glycosylphosphatidylinositol was linked to a disorder characterized by psychomotor retardation and facial dysmorphism. Whole exome sequencing (WES) was performed in siblings with severely delayed myelination and psychomotor retardation. Mutations in *PGAP1* were confirmed by Sanger sequencing and RNA analysis. A literature search was performed to describe the emerging phenotype of *PGAP1* related disease.

**Case presentation:**

WES resulted in the detection of two novel compound heterozygous mutations in *PGAP1*, one base pair insertion leading to a frame shift c.334_335InsA (p.A112fs) and a splice site mutation leading to exon skipping c.G1173C (p.L391L). A symptom not described in *PGAP1* related disorder before but prominent in the siblings were recurrent apnea especially during sleep that persisted at least until age 2 years. Sequential cerebral MRI at age one and two year(s) respectively revealed frontal accentuated brain atrophy and significantly delayed myelination.

**Conclusion:**

We report siblings with two novel mutations in *PGAP1*. Other that the common symptoms related to *PGAP1* mutations including non-progressive psychomotor retardation, neonatal feeding problems, microcephaly and brain atrophy these patients displayed severely delayed myelination and recurrent apneas thereby widing the clinical spectrum associated with such mutations.

**Electronic supplementary material:**

The online version of this article (doi:10.1186/s12883-016-0602-7) contains supplementary material, which is available to authorized users.

## Background

Genetic defects that affect the proper synthesis and remodeling of glycosylphosphatidylinositol (GPI) anchors of cell surface proteins are a new group of autosomal recessive disorders leading to intellectual disability. Of the 26 genes that are involved in this process, mutations in 13 have been described [1]. Other than intellectual disability, congenital malformations affecting the central nervous system (CNS) and other organs have been described [2]. *PGAP1* codes for PGAP1 (post-GPI attachment to proteins 1) that is responsible for the first step of the remodeling of glycosylphosphatidylinositol (GPI)-anchors after they have been transferred to the proteins by the GPI transaminase. PGAP1 is an endoplasmic reticulum (ER)-associated GPI inositol-deacylase, which cleaves the acyl chain from the inositol [3]. In 2014 the first mutations, homozygous deletions c.589_591delCTT (p.Leu197del), in *PGAP1* were described in siblings from Syria, floppy infants with developmental delay and severe intellectual disability. Murakami et al. demonstrated that the defect of PGAP1 did not lead to a decreased expression level of GPI-anchored proteins on B lymphoblastoid cells derived from one of the subjects, but to GPI-anchors with an abnormal structure and altered biochemical properties [2]. Since this original description 4 more families with mutations in this gene have been described, all of them a result of whole-exome sequencing (WES). Novarino et al. used WES to identify the genetic base of the disease in 55 families with hereditary spastic paraplegias [4]. They discovered a homozygous splice site mutation (c.1952 + 1G > T) in *PGAP1* in two male first degree cousins with global developmental delay, abnormal hand movements and increased deep tendon reflexes suggesting a motor neuron disease. More recently three further families have been described. Firstly a 3 year old boy from the United States with developmental delay and cortical visual impairment who was found to be compound heterozygous for two nonsense variants, c.1572 T > A (p.Tyr524*) and c.1396C > T (p.Gln466*) in *PGAP1* [5]. Secondly an 8 years old boy from unrelated parents with muscular hypotonia, feeding difficulties and developmental delay who was shown to carry compound heterozygous c.274_276del (p.Pro92del) and c.921_925del (p.Lys308Asnfs*25) mutations in the *PGAP1* gene [6]. Thirdly siblings of a consanguineous couple from North Turkey who are 1st cousins, with muscular hypotonia, severe developmental delay, microcephaly and retinal dystrophy carrying a homozygote splice site mutation c.1090-2A > G [7].

In this article we show the clinical symptoms and MRI findings of two further patients with mutations in *PGAP1* thereby confirming and extending the phenotype of *PGAP1* related disease.

## Case presentation

### Clinical phenotype

**Fig. 1:**
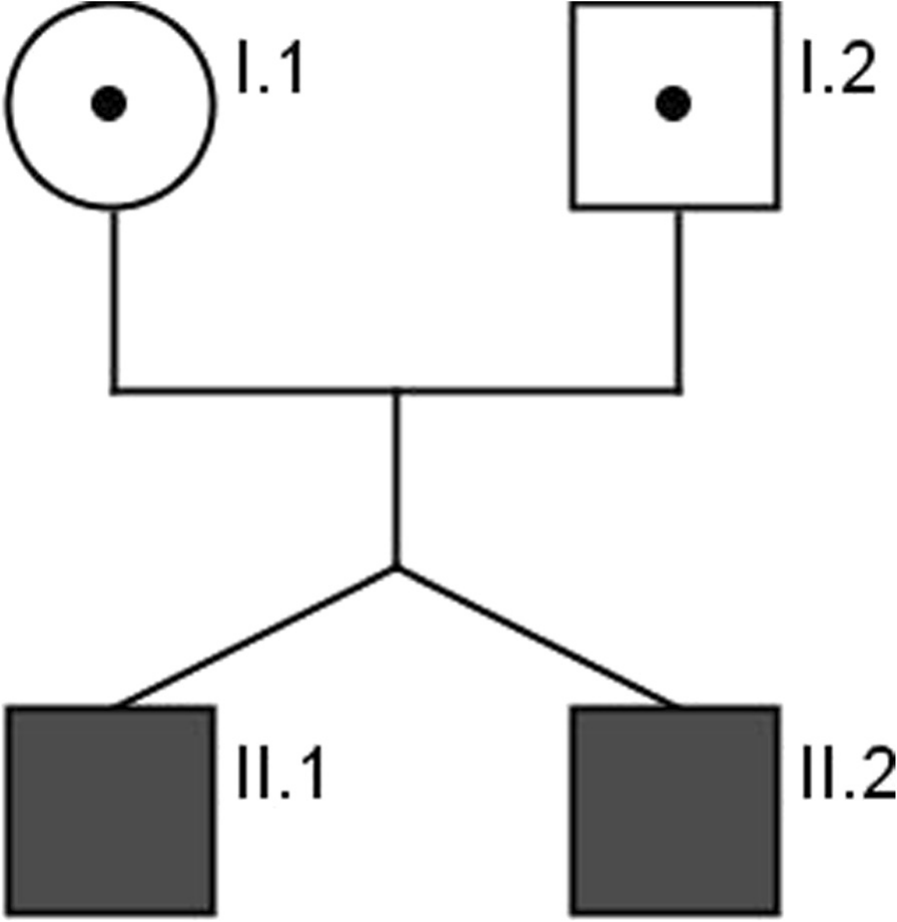
Pedigree of reported family. Pedigree chart designer by CeGaT GmbH [11]

Patients II-1 and II-2, dizygotic male twins and first children of non-consanguineous Caucasian parents with an unremarkable family history were born in the 30^th^ week of gestation due to maternal HELLP syndrome (Fig. 1). Birth measures were normal for gestational age (II-1: weight 1555 g, length 43 cm; II-2: weight 1410 g, length 41 cm) and no congenital anomalies were present. Both children developed respiratory distress syndrome and were treated with surfactant. At age 3 months recurrent prolonged apnea occurred necessitating stimulation mainly during sleep. Multiple EEGs did not show epileptic activity during these episodes that persisted until age 2 years when they were last seen, despite anticonvulsive therapy. Psychomotor development was severely delayed in both children. At a corrected age of 12 months they showed pendular eye movements with rotation nystagmus, no fixation or tracking. Generalized muscular hypotonia was present with poor head control and paucity of spontaneous movements. Increased tendon reflexes with positive Babinski sign were noted as well as frequent myoclonus and tremor. At age 25 months II-1 was able to roll over and momentarily sit unsupported. He was very alert and friendly, fixated and played with objects. He could babble but did not speak. He showed frequent myoclonus and a pronounced hyperexcitability. Patient II-2 developed epilepsy during the 2^nd^ year of life with atone seizures, desaturation, gaze deviation and smacking movements that were successfully treated with topiramate. At age 25 months he showed less developmental progress than his brother. He was able to roll on the side and grasp objects but did not interact with the caretakers. Like his brother he showed myoclonus and was hyperexcitable. At age 2 years both children presented with normal height and length. Head circumference was on the 3^rd^ centile in patient II-1 and below the 3^rd^ centile in patient II-2.

**Fig. 2:**
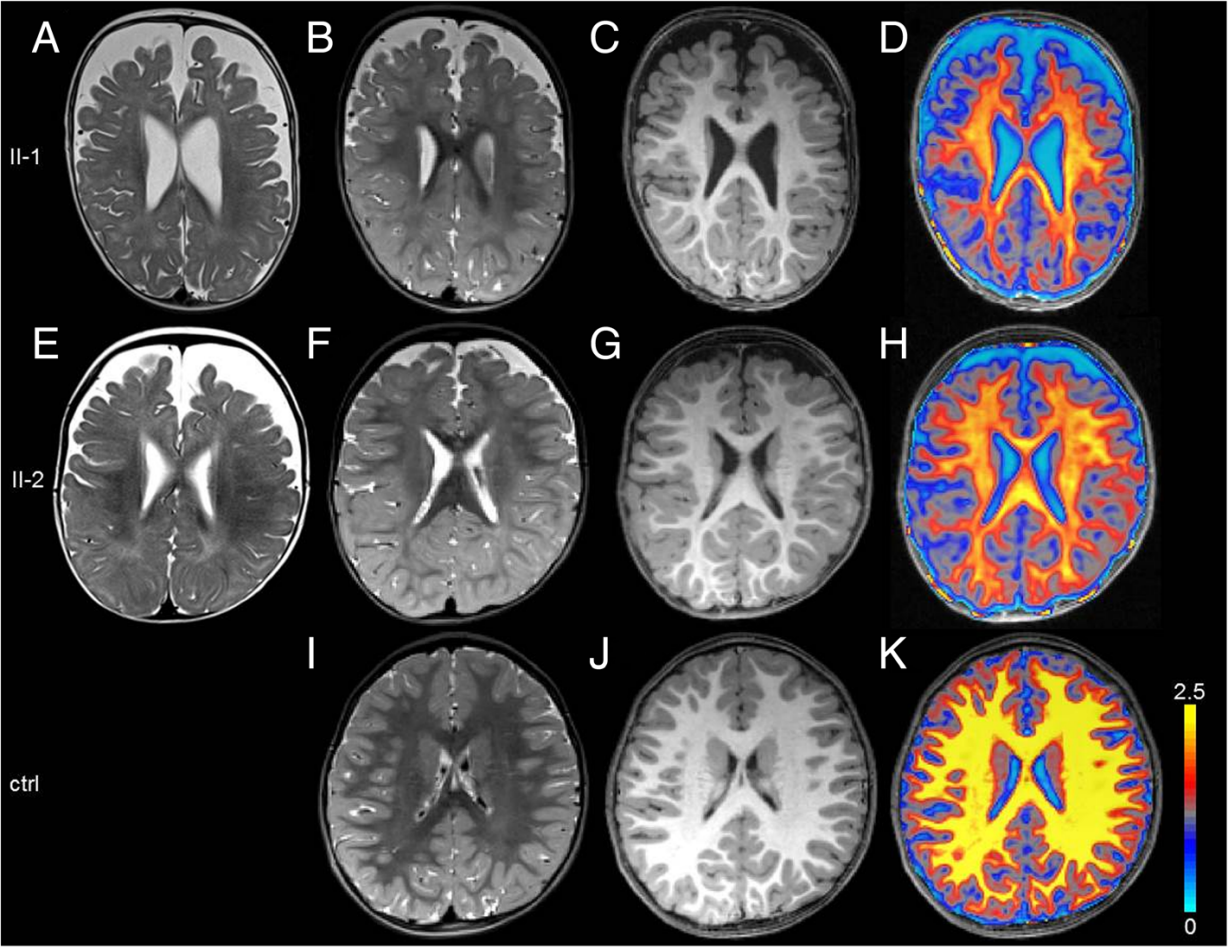
Structural and quantitative MRI of patients and a healthy age matched control. **a**;**b**;**e**;**f**;**i**: T2-weighted images at the corrected age of 12 months (**a**, **e**) and 2 years (**b**, **f**, **i**) of patients II-1 (**a**, **b**) and patient II-2 (**e**, **f**) and control (**i**) showing brain atrophy and significant delayed myelination in the patients. **c**;**d**;**g**;**h**;**j**;**k**: T1-weighted images and overlay of color coded MT saturation maps onto the corresponding T1-weighted images of both patients (**c**, **d**, **g**, **h**) and an age matched control (**j**, **k**) at age 2 years. Yellow represents high signal intensity considered to arise from properly myelinated WM. Both patients show widespread marked reductions of the myelinsensitive parameter MT saturation [12] when compared to the age matched control thereby emphasizing the delayed myelination. For detailed information about MT saturation maps see Additional file 1

**Fig. 3:**
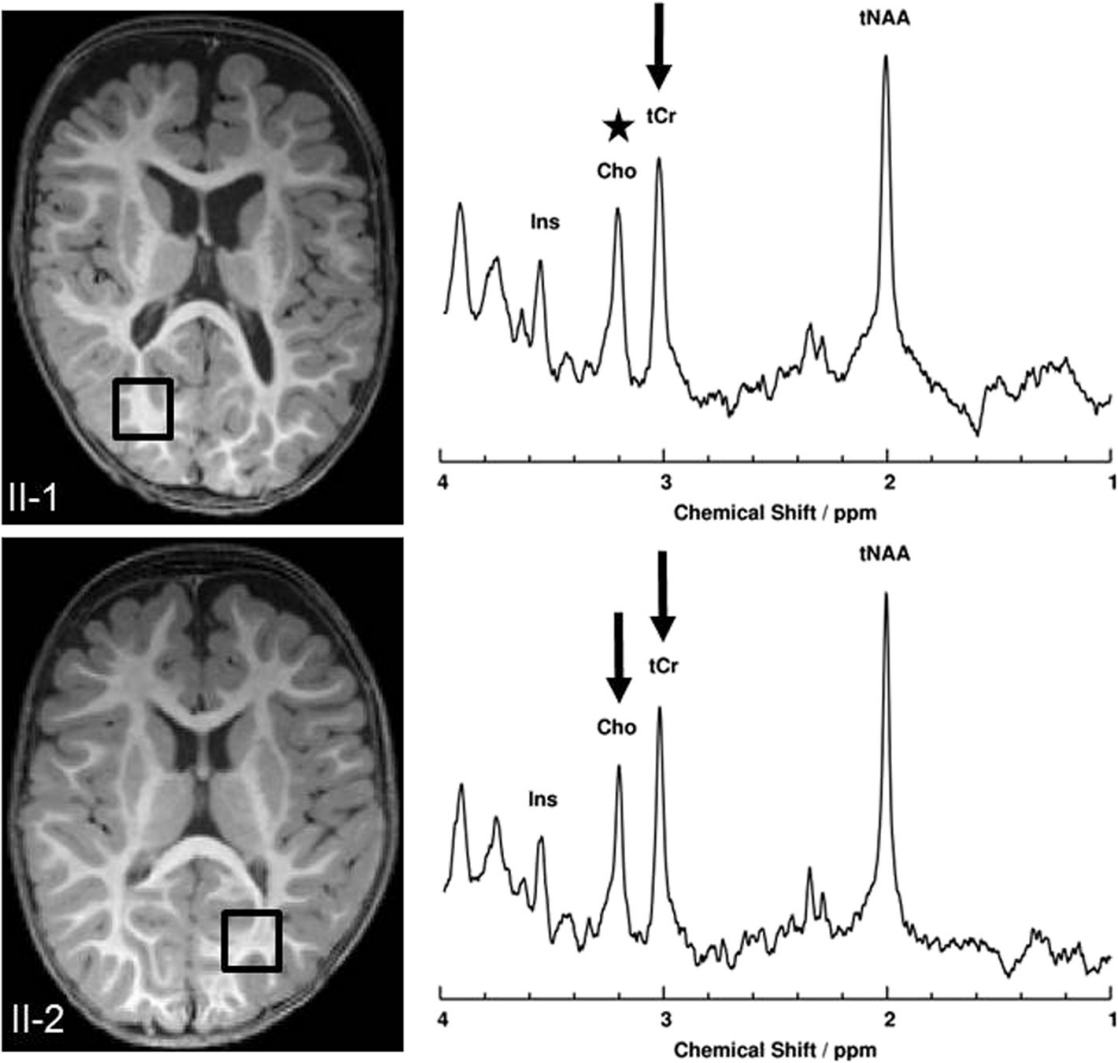
Structural MRI and MR-Spectroscopy. MR-Spectroscopy of parieto-occipital WM from both patients. Placements of volume of interest are illustrated in T1- weighted images (*left*). Significant decreases (>2 SD of mean control value) of metabolite concentrations are indicated by black arrows. The black asterix indicates marginally low concentration. (tNAA: sum of N-acetylaspartate and N-acetylaspartylglutamate, tCr: sum of creatine and phosphocreatine, Cho: Choline, Ins: Inositol). For detailed information about MR-Spectroscopy see Additional file 1

Investigations for metabolic disorders including serum levels for alkaline phosphatase, calcium, parathyroid hormone and vitamin D, that have been found abnormal in patients with other defects in the synthesis of GPI-anchors, were repeatedly normal [8]. Nerve conduction velocity as well as acoustic and visual evoked potentials measured in patient II-2 were within the normal range. Cerebral MRI at age one year showed frontal accentuated brain atrophy and significantly delayed myelination in T2-weighted images. On follow-up MRI at age two years the amount of myelin was increased, however the magnetization transfer (MT) saturation maps still revealed a striking reduction of signal arising from myelin compared to age matched controls. Brain atrophy was also less pronounced in the follow up MRI (Fig. 2). MR spectroscopy at age two years demonstrated a significant reduction (>2 SD of mean control values [9]) of creatine and choline concentration in both patients which may point to a decreased amount of choline containing myelin proteins and cell density a finding that has been described in hypomyelinating conditions [10] (Fig. 3).

### Molecular Analysis

The exome analyses yielded 65.96 million confidently mapped reads with a mean coverage of X69. Following alignment to the reference genome, 21,931 on-target variants were noted. We removed variants which were called less than X8, were off-target, synonymous (>4 bp from exon/intron junction), MAF > 0.1 % at dbSNP138 and MAF > 1 % in the Hadassah in-house dbSNP. 98 variants survived this filtering process but only a single couple resided in the same gene, *PGAP1*. These were chr2: 197781284InsT, NM_024989:c.334_335insA:p.A112fs and Chr2: 197755552 C > G, NM_024989:c.G1173C:p.L391L, affecting the donor splice site of exon 10.

**Fig. 4:**
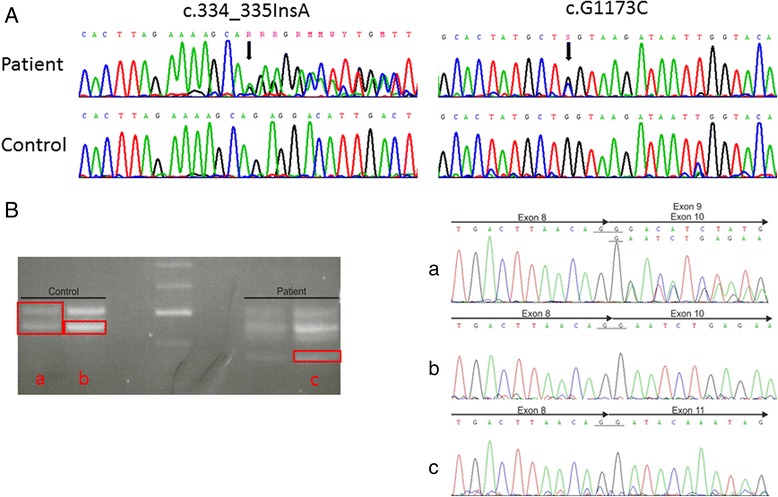
Sanger sequencing and cDNA analysis. **a**: Sanger sequencing of the *PGAP1* gene in patient II-1 (upper row) and a control (lower row). The arrows indicated the compound heterozygous mutations. **b**: Agarose gel electrophoresis of cDNA from blood of Patient II-1 on the right and healthy control on the left. The red rectangles indicate the eluted bands using for subsequently sequencing. Letter a, b and c refer to the corresponding sequences on the right

Sanger sequencing showed that both patients were compound heterozygous for the insertion, c.334_335InsA (p.A112fs) which was shown to be inherited from the mother, leading to a frame shift and the splice site mutation, c.G1173C (p.L391L), in the *PGAP1* gene which was shown to be inherited from the father. cDNA analysis from whole blood of patients and a healthy control revealed on the one hand a transcript variant without exon 9 in patients as well as in healthy control. Additionally the splice site mutation c.G1173C (p.L391L) leads to skipping of exon 10 (Fig. 4). Thereby it is to suppose that the skipping of the exon 10 leads to a truncated protein.

For detailed information about WES and Sanger sequencing see Additional file 1.

## Discussion

Including the two patients reported on in this article 10 patients from 6 different families carrying mutations in the *PGAP1* gene have been described so far [2, 4–7]. A summary of the clinical and genetic findings is given in Table 1. In none of the patients the disorder had a progressive course instead the patients showed a varying degree of development. 8 patients were reported to have had muscular hypotonia in the neonatal period leading to feeding problems in 6. Tremor was observed in 3 patients, dyskinetic movements in 6. Only two patients have been reported to walk independently, however the majority of the patients were still young. Intellectual disability seems to be a constant feature but with varying severity. Only one patient has been reported to be able to speak. This patient was also the only normocephalic patient while the others were microcephalic indicating that he might be less severely affected. Epilepsy was present in three out of ten patients and one additional patient showed generalized slowing in the EEG without clear epileptiform changes. Recurred apneas especially during sleep, which were a very prominent symptom in the patients described in this article, have not been described in the other patients. Dysmorphic features described in the patients include large ears, a flattened nasal root [2], prominent forehead, large mouth, abnormal teeth, high arched eyebrows, a short neck [5], upward slanting palpebral fissures, deep-set eyes, large ear lobes and prominent helices and antihelices, extra mamelons of teeth and diminished enamel [6]. In addition to the face malformations Williams et al. described abnormal hand morphology in his patient. Metabolic analysis in the *PGAP1* patients has overall yielded unremarkable results. Hyperphosphatasia that was described in other GPI-APs related disease (PIGV, PIGO and PGAP2) due to recognition of mannose residue in immature GPI and the consecutive cleavage of the hydrophobic signal peptide from the proproteins by GPI transamidase resulting in secretion of soluble alkaline phosphatase [8]. But serum levels of alkaline phosphatase were normal in our patients and in the *Pgap1* mouse model as well, suggesting elevated alkaline phosphatase is not part of *PGAP1* disease. Imaging of the brain was performed in 9 out of 10 patients (1 CT, 8 MRIs). Cerebral imaging revealed global brain atrophy in 5/9 patients, defective myelination in 4/9, abnormalities of the sylvian fissure in 2/9 and callosum agenesis and vermis hypoplasia in one. One patient, the only patient that was able to speak, was reported to have a normal MRI at the age of 5 years again supporting the milder course of the disease in this patient. The MRIs of the siblings from North Turkey were also described to be normal, but information of the patients’ age at investigation was not provided. Sequential MRIs, available in the patient described by Williams et al. and the two patients reported on in this article, demonstrate that the amount of myelin detected on MRI is increasing indicating delayed myelination rather than defective myelination. However, the analysis of the MT saturation maps shows that the amount of myelin is still decreased when compared to age matched controls at the age of 2 years. Single voxel proton MR spectroscopy also indicated a defect in the myelin.

**Table 1:** Clinical and genetic findings of *PGAP1*-disease

	This Study		Murakami et al.		Bosch et al.	Williams et al.	Novarino et al.		Granzow et al.	
	II-1	II-2	III-2	III-3			IV-3	IV-4	I-1	I-2
Family history	Dizygotic Caucasian twins of non-consanguineous parents		Sibling of consanguine Syrian relationship		non-consanguineous	non-consanguineous	consanguineos/1° cousins		consanguineos/1° cousins	
Genotype	c.334_335InsA (p.Arg112fs)		homozygous c.589_591del (p.Leu197del)		c.274_276del (p.Pro92del)	c.1572 T > A (p.Tyr524*)	homozygous splice site mutation c.1952 + 1G > T		homozygous splice site mutation c.1090-2A > G	
	AND				AND	AND				
	splice site mutation c.1173G > C (p.Leu391Leu)				c.921_925del (p.Lys308Asnfs*25)	c.1396C > T p.(Gln466*)				
Gender	M	M	F	M	M	M	M	M	M	F
Age^a^	2	2	4 5/12	2 9/12	7 10/12	3	6 6/12	9/12	8	4
Pregnancy/Birth	30th weeks, maternal HELLP syndrome		unremarkable		planned CS, 38 weeks	39 weeks	NA	NA	unremarkable	
Facial dysmorphisms	(+)	(+)	+	+	+	+	-	-	-	-
Microcephaly	+	+	+	+	-	+	NA	NA	+	+
Intellectual disability	+	+	IQ < 35	IQ < 35	IQ 49	+	+	-	+	+
Speech	Babble	Babble	Babble	NA	2 6/12	delayed	NA	NA	-	-
Motor developmental delay	+	+	+	+	+	+	+	-	+	+
Walking independently (years)	-	-	4 5/12	-	2 6/12	-	NA	NA	NA	-
Hypotonia	+	+	+	+	+	+	NA	NA	+	+
Neuropathy/Spasticity	-	-	NA	NA	-	-	+	+	-	+
Stereotypic/dyskinetic movements	-	-	+	+	-	+	+	+	+	-
Eating/Feeding	Milk bottles	Milk bottles	Milk bottles	Milk bottles	-	G-tube feeding	NA	NA	-	Failure to thrive
Respiration	Recurrent apneas	Recurrent apneas	-	-	-	-	NA	NA	+	-
Ophthalmological findings	-	-	-	-	CVI	CVI	-	-	Retinal dystrophy (ERG)	
Seizures/EEG abnormalities	−/−	+/+	+/NA	-/NA	-/NA	−/+	NA	NA	−/−	−/−
Imaging	Frontal accentuated brain atrophy and significant delayed myelination (MRI)		Brain atrophy (CT)	NA	Normal (MRI)	Thinning of thecorpus collosum, diminished white matter, prominence of the right posterior Sylvian fissure (MRI)	Prominent cortical sulci and widened sylvian fissures (MRI)	Corpus callosum agnesis, vermis hypoplasia, defective myelination (MRI)	Normal (MRI)	Normal (MRI)

*CS* caesarian section, *CT* computer tomography, *CVI* cortical visual impairment, *EEG* electroencephalogram, *ERG* electroretinogram, *F* female, *IQ* intelligence quotient, *M* male, *MRI* magnetic resonance imaging, *NA* not available

^a^ Age at investigation

A knock-out mouse model of *PGAP1* was described in 2007 with three different phenotypes that varied in severity. Some mice showed a severe developmental defect of the brain, similar to human otocephaly and others only minor abnormalities like growth retardation or male infertility. The mouse model therefore seems to resemble some of the features of *PGAP1* related human disease including the wide phenotypic variability.

## Conclusion

The emerging clinical phenotype of *PGAP1* related disease is rather unspecific. It includes non-progressive severe psychomotor retardation and infrequent mild facial dysmorphism. The probably most specific finding for *PGAP1* related disease, at least in our patients, was severely delayed myelination. The fact that within only one year since the original description 8 more patients with genetic defects of *PGAP1* have been described might indicate that such defects are a more common cause of intellectual disability and that the *PGAP1* gene should be included in gene panels for the analysis of severe psychomotor retardation and delayed myelination.

### Ethics approval and consent to participate

Ethics approval is not applicable. Informed consent to all diagnostic procedures was given by the caregivers.

### Consent for publication

Written informed consent for publication of their clinical details and MRI images was obtained from the parents of the patients. A copy of the consent form is available for review by the Editor of this journal.

### Availability of data and materials

A detailed description of all use methods is given in Additional file 1.

## Additional file

Additional file 1: Methods – Description of Quantitative magnetization transfer (MT) imaging, proton MR-spectroscopy, Whole exome analysis, RNA isolation and cDNA synthesis. (DOCX 19 kb)

## Abbreviations

cDNA: complementary DNA
CNS: central nervous system
DNA: deoxyribonucleic acid
EEG: electroencephalography
ER: endoplasmic reticulum
GPI: glycosylphosphatidylinositol
GPI-AP: glycosylphosphatidylinositol-anchored protein
HELLP syndrome: acronym for a syndrome with Hemolysis Elevated Liver enzymes and Low Platelet count
MAF: minor allele frequency
MR: magnetic resonance
MRI: magnetic resonance imaging
MT: magnetization transfer
tCr: creatine and phosphocreatine
tNAA: *N*-acetylaspartylglutamate
WES: whole-exome sequencing
WM: white matter
