# Supplementary material for: Compound heterozygous variants in PGAP1 causing severe psychomotor retardation, brain atrophy, recurrent apneas and delayed myelination: a case report and literature review
Source: BMC Neurol. 2016 May 21;16:74. doi: 10.1186/s12883-016-0602-7 (PMC4875637; doi:10.1186/s12883-016-0602-7)
Supplement: Additional file 1: — Methods – Description of Quantitative magnetization transfer (MT) imaging, proton MR-spectroscopy, Whole exome analysis, RNA isolation and cDNA synthesis. (DOCX 19 kb) [file 12883_2016_602_MOESM1_ESM.docx]

**Methods**

**Quantitative magnetization transfer (MT) imaging**

In addition to the conventional neuroradiological MR examination a quantitative myelinsensitive MT measurement was performed according to our established MR-protocol for white matter disorders in childhood [8]. A 3T clinical MR system (Tim Trio, Siemens Healthcare, Erlangen, Germany) was employed for all MR studies. The protocol comprised a 3D FLASH (fast low angle shot) sequence 1.25 mm isotropic resolution and 240 mm field-of-view. MT contrast was imposed upon a proton density (PD)-w reference (repetition time (TR)/ echo time (TE)/flip angle α = 25/4.9 ms/5°) by applying a 12.8 ms Gaussian MT-pulse (540° nominal flip angle 2.2 kHz off resonance). By means of a second T1-w reference (TR/α=11 ms/15°, 1.5 minutes), maps of the percentage MT saturation were calculated. Data processing was scripted using the routines of the FSL 4.1 software library of the Centre for Functional Magnetic Resonance Imaging of the Brain (FMRIB, Oxford, UK). The cyan-blue-gray-red-yellow color scale of the MT saturation maps covered a range from -0.1 pu (cyan; cerebrospinal fluid (CSF)) to 1.2 pu (gray, gray matter (GM)) to 2.5 pu (yellow; white matter (WM)). Myelinated WM of controls (MT saturation > 2.5 pu) appeared uniformly yellow. Red indicates partial volume of WM and GM. Dark blue indicates edema or partial volume of CSF and parenchyma [9].

**Proton MR-spectroscopy**

Stimulated echo acquisition mode (STEAM) sequence was used to acquire single voxel spectra (64 accumulations, TR/TE/mixing time = 6000/20/10 ms). Placement of the volume-of-interest (4.1 ml) is indicated in figure 2. Absolute concentrations of the neuroaxonal marker N-acetylaspartate and N-acetylaspartylglutamate (tNAA), creatine and phosphocreatine (tCr) correlated with energy status and cell density, choline-containing compounds (Cho) related to myelin turnover and the astrocytic marker inositol (Ins) were determined by LCModel [10] and compared to age-matched controls (own data base and [11]).

**Whole exome analysis**

Exonic sequences were enriched in the DNA samples of the patient using SureSelect Human All Exon 50 Mb Kit (Agilent Technologies, Santa Clara, California, USA). Sequences were determined by HiSeq2000 (Illumina, San Diego, California, USA) and 100-bp were read paired-end. Reads alignment and variant calling were performed with DNAnexus software (Palo Alto, California, USA) using the default parameters with the human genome assembly hg19 (GRCh37) as a reference. Parental consent was given for DNA studies.

**RNA isolation and cDNA synthesis**

RNA was isolated from whole blood using the “PAXgene Blood RNA System” (PreAnalytiX, PreAnalytiX GmbH, Hombrechtikon, Switzerland). Extracted RNA was stored at -80°C until usage. Complementary DNA was synthesized by use of SuperScript III Reverse Transcriptase with Oligod(T) primer (Life Technologies, Darmstadt, Germany). The detection of the skipped exon was performed by PCR (Quiagen GmbH, Hilden, Germany) using the following primers: forward: 5’ CTC TCC ATT GTG TGG TGT AAA C 3’ and 5’ TAC AAC AAA CTT ACT TCC ACG AAC 3’. For sequencing, specific bands were eluted from the gel by use of NucleoSpin® Gel and PCR Clean-up (Machery-Nagel, Düren, Germany) and subsequently processed for direct dye terminator sequencing with BigDye Terminator Ready Reaction chemistry 3.1 on an ABI PRISM 3130-Avant genetic analyser (Life Technologies, Darmstadt, Germany). All reactions were performed according to instructions provided by the manufacture.

**List of abbreviations**

Cho: Choline-containing compounds

CSF: Cerebrospinal fluid

DNA: Deoxyribonucleic acid

GM: Gray matter

Ins: Inositol

MR: Magnetic resonance

MRI: Magnetic resonance imaging

MT: Magnetization transfer

PCR: Polymerase chain reaction

STEAM: Stimulated echo acquisition mode

tCr: Creatine and phosphocreatine

TE: Echo time

tNAA: N-acetylaspartylglutamate

TR: Repetition time
